# Something Old, Something New: Ion Channel Blockers as Potential Anti-Tuberculosis Agents

**DOI:** 10.3389/fimmu.2021.665785

**Published:** 2021-06-24

**Authors:** Steven C. Mitini-Nkhoma, Elizabeth T. Chimbayo, David T. Mzinza, David V. Mhango, Aaron P. Chirambo, Christine Mandalasi, Agness E. Lakudzala, Dumizulu L. Tembo, Kondwani C. Jambo, Henry C. Mwandumba

**Affiliations:** ^1^ Malawi-Liverpool-Wellcome Trust Clinical Research Programme, University of Malawi College of Medicine, Blantyre, Malawi; ^2^ Department of Clinical Sciences, Liverpool School of Tropical Medicine, Liverpool, United Kingdom

**Keywords:** mycobacterium, tuberculosis, host-directed therapies, ion channel blocker, efflux pump, drug-repurposing

## Abstract

Tuberculosis (TB) remains a challenging global health concern and claims more than a million lives every year. We lack an effective vaccine and understanding of what constitutes protective immunity against TB to inform rational vaccine design. Moreover, treatment of TB requires prolonged use of multi-drug regimens and is complicated by problems of compliance and drug resistance. While most *Mycobacterium tuberculosis* (Mtb) bacilli are quickly killed by the drugs, the prolonged course of treatment is required to clear persistent drug-tolerant subpopulations. Mtb’s differential sensitivity to drugs is, at least in part, determined by the interaction between the bacilli and different host macrophage populations. Therefore, to design better treatment regimens for TB, we need to understand and modulate the heterogeneity and divergent responses that Mtb bacilli exhibit within macrophages. However, developing drugs *de-novo* is a long and expensive process. An alternative approach to expedite the development of new TB treatments is to repurpose existing drugs that were developed for other therapeutic purposes if they also possess anti-tuberculosis activity. There is growing interest in the use of immune modulators to supplement current anti-TB drugs by enhancing the host’s antimycobacterial responses. Ion channel blocking agents are among the most promising of the host-directed therapeutics. Some ion channel blockers also interfere with the activity of mycobacterial efflux pumps. In this review, we discuss some of the ion channel blockers that have shown promise as potential anti-TB agents.

## Introduction

Tuberculosis (TB) is an airborne infection contracted by inhalation of droplet nuclei containing viable *Mycobacterium tuberculosis* (Mtb) that are released into the air by a person with active pulmonary TB. The disease has been a major cause of morbidity and mortality for several millennia (1). In 2019 alone, 10 million people developed active TB and 1.4 million of them died of the disease (2). Most of the TB cases in 2019 were in South-East Asia (44%), Africa (25%) and western Pacific (18%) (2).

TB is challenging to treat even though there are now more than 20 first- and second-line anti-TB drugs in clinical use (3). Current anti-TB treatment regimens utilize combinations of no less than 3 drugs that must be taken for at least 6 months (3). The lengthy treatment duration and side effects of the drugs often lead to poor compliance with treatment, unfavorable outcomes and development of drug-resistant Mtb strains (4). In 2019, more than 0.5 million people developed multidrug-resistant (MDR) or rifampicin (RIF)-resistant (RR) TB worldwide (2). Treatment of drug-resistant TB requires longer and more complex drug regimens, and often causes more serious adverse effects than treatment of drug-susceptible TB (5). Current TB drugs target the pathogen and function by compromising the structural integrity or metabolic machinery of Mtb. In the last few years, host-directed therapy (HDT) targeting macrophages has emerged as a promising therapeutic strategy for both drug-susceptible TB and MDR-TB.

In the lung, alveolar macrophages (AMs) are among the most important innate defenses against Mtb. They phagocytose and eliminate bacteria through various pathways including phagosome maturation, autophagy and apoptosis. However, Mtb has evolved to survive inside macrophages by corrupting macrophage antimicrobial responses. HDTs for TB aim to rectify or circumvent the corrupted antimycobacterial responses.

**Figure 1:**
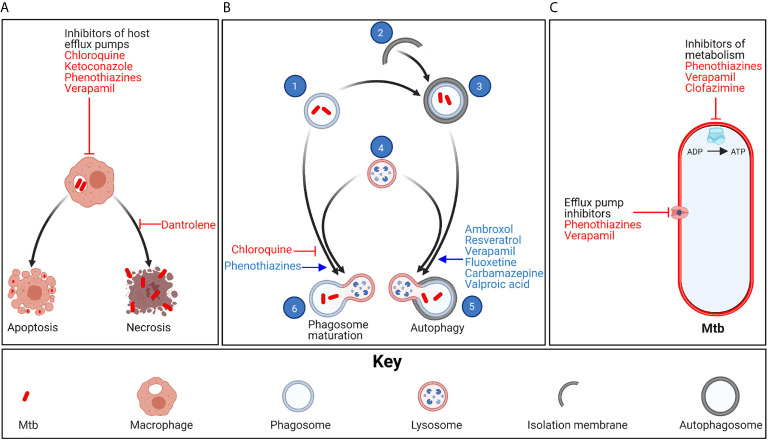
Mechanism of action of ion channel blockers. **(A)** Chloroquine, ketoconazole, phenothiazines and verapamil inhibit eukaryotic efflux systems, allowing anti-TB drugs to achieve higher concentrations inside Mtb-infected host cells. Mtb promotes necrotic death of infected macrophages, leading to release of the bacteria into the extracellular space, where the bacteria continue to proliferate in the necrotic cells or infect new cells. In contrast, apoptotic cell death leads to enzymatic degradation of most of the bacteria. Dantrolene prevents necrotic death of Mtb-infected macrophages. **(B)** Phagocytosed Mtb (1) can also be eliminated through phagosome maturation and autophagy. In autophagy, isolation membranes (2) elongate and engulf phagosomes. The autophagosome-sequestered phagosomes (3) then fuse with lysosomes (4) to form autophagolysosomes (5), following which the lysosomal enzymes degrade the phagocytosed bacteria. Ambroxol, resveratrol, verapamil, fluoxetine, carbamazepine and valproic acid promote autophagy. In phagosome maturation, Mtb phagosomes fuse with lysosomes to form phagolysosomes (6). Phenothiazines promote acidification of phagolysosomes, thus enhancing activity of the lysosomal enzymes. In contrast, chloroquine inhibits phagosome maturation, thus preventing redox-induced Mtb drug tolerance, making the bacteria more susceptible to anti-TB drugs. **(C)** Phenothiazines and verapamil can also inhibit Mtb metabolism and efflux pump activity. Clofazimine, a second-line anti-TB agent, also inhibits Mtb metabolism. Created with BioRender.com.

Ion channel blockers are among the most promising potential HDTs for TB (**Table 1**). They are a diverse group of compounds that alter cell physiology by attenuating ion currents across cellular and subcellular membranes, and are most commonly used to treat noncommunicable diseases such as hypertension. Several Food and Drug Administration (FDA)-approved ion channel blocking agents have shown promise at both enhancing Mtb clearance by the immune system and attenuating inflammation *in vitro* and in animal models of TB (**Figure 1**). Additionally, some ion channel blocking agents have direct antimycobacterial activity. Here we review ion channel blocking agents that have demonstrated anti-tuberculosis activity in Mtb-infected macrophages and/or in animal models of TB.

**Table 1:** Progress towards clinical use of ion channel blockers as anti-tuberculosis agents.

Year	Milestone	Reference
1990	Crowle and May demonstrated that chloroquine inhibits Mtb growth in macrophage cultures and potentiates streptomycin, pyrazinamide and isoniazid	(6)
1992	Crowle and colleagues observed that chlorpromazine was more active against Mtb in macrophage cultures than in broth	(7)
1993	Klemens and colleagues reported that clofazimine was effective against an MDR-TB strain in mice	(8)
1994	Gollapudi and colleagues demonstrated that verapamil improves accumulation of INH in Mtb-infected macrophages and promotes sensitivity of Mtb to INH	(9)
1996	Grange and Snell demonstrated that ambroxol has antimycobacterial activity in macrophages	(10)
2003	Esiobu and Hoosein observed that sodium valproate inhibits growth of *Mycobacterium smegmatis* in broth	(11)
2007	Byrne and colleagues observed that ketoconazole was synergistic with rifampicin-isoniazid-pyrazinamide	(12)
2010	van Deun and colleagues successfully used clofazimine as part of a 9-month MDR-TB treatment regimen in a clinical trial	(13)
2013	Smolarz and colleagues demonstrated that resveratrol has antitubercular activity in broth	(14)
2014	Stanley and colleagues demonstrated that fluoxetine promotes autophagic control of Mtb in macrophages	(15)
2015	Schiebler and colleagues successfully reduced the bacteria burden in mice infected with MDR-TB using carbamazepine and valproic acid	(16)
2016	Machado and colleagues successfully used verapamil, thioridazine and chlorpromazine to decrease bacteria burden in Mtb-infected macrophages	(17)
2016	WHO conditionally recommended a short course MDR-TB treatment regimen containing clofazimine	(18)
2018	Choi and colleagues demonstrated that ambroxol promotes autophagy and potentiates rifampicin in murine models of TB	(19)
2018	Rao and colleagues demonstrated that sodium valproate has antimycobacterial activity in broth and in macrophages in culture, and enhances activity of rifampicin and isoniazid	(20)
2019	Roca and colleagues demonstrated that dantrolene inhibits necrotic death and promotes Mtb control in Mtb-infected macrophages	(21)
2019	Yang and colleagues demonstrated that resveratrol has antitubercular activity in mice	(22)
2021	Lee and colleagues observed that the use of calcium channel blockers was associated with a 32% decrease in the risk of active tuberculosis	(23)

## Ion Channel Blockers With Potential as Anti-Tuberculosis Agents

### Calcium Channel Blockers

Calcium ions (Ca^2+^) act as second messengers in several signal transduction pathways (24). Calcium is more abundant in the extra cellular fluid (ECF) than in the cytosol (25). In the cell, most of the Ca^2+^are sequestered in endoplasmic reticuli (ER) (25). Cell activation signals induce the flow of Ca^2+^ from the ER and ECF into the cytosol through channels such as inositol-1,4,5-trisphosphate receptors (IP_3_Rs), ryanodine receptors (RyRs), voltage-gated calcium channels (VGCC) and calcium release-activated calcium (CRAC) channels.

Ca^2+^ signaling is important in antimycobacterial responses, including autophagy, phagosome maturation and apoptosis. In general, high cytosolic concentrations of Ca^2+^ promote phagosome maturation and acidification of mycobacteria-containing phagosomes, necrosis and apoptosis; while cell autophagy can be both upregulated or downregulated by Ca^2+^ (26–28). Whether Ca^2+^ influx upregulates or downregulates autophagy depends on factors such as the biological context and the ion channel conducting the Ca^2+^ current. For example, Ca^2+^ currents through the ATP-gated cation channel P2X_7_ receptor enhanced autophagy and intracellular killing of *M. bovis*-BCG in human macrophages, while currents through VGCCs inhibit autophagy (26, 28).

A recent population-based analysis investigated whether the use of calcium channel blockers modifies the risk of active TB among patients with heart failure or cerebrovascular diseases in the clinical setting (23). The analysis included 8164 new active TB patients and 816,400 controls treated with or without calcium channel blockers compared with β-blockers or loop diuretics. Overall, the use of calcium channel blockers was associated with a 32% decrease in the risk of active TB [relative risk (RR), O.68 (95% CI, 0.58-0.78)] after adjustment with disease risk score. Analysis of the effect of different types of calcium channel blockers revealed that use of dihydropyridine calcium channel blockers was associated with a lower risk of TB [RR, 0.63 (95% CI, 0.53-0.79)] than non-dihydropyridine calcium channel blockers [RR, 0.73 (95% CI, 0.54-0.94)]. β-blockers or loop diuretics were not associated with lower risk of TB [RR, 0.99 (95% CI, 0.83-1.12)] and [RR, 0.88, (95% CI. 0.62-1.26)], respectively (23). This is the first large population-based study to confirm that calcium channel blockers modify and reduce the risk of active TB in humans. Therefore, modulating Ca^2+^ signaling using calcium channel blockers is an attractive host-directed therapeutic strategy for TB. Dantrolene, resveratrol and verapamil are the calcium channel blockers that have shown the most promise as potential anti-TB agents.

#### Dantrolene

Dantrolene is a RyR antagonist clinically approved for treatment of malignant hyperthermia. RyRs are intracellular Ca^2+^ channels that mediate the release of Ca^2+^ from the ER in response to elevated cytosolic Ca^2+^ levels (29). They are important in physiological and pathological processes, including necrosis and apoptosis. Apoptosis involves enzymatic degradation of intracellular contents including most of the phagocytosed bacteria, and their packaging into fragments called apoptotic bodies (30, 31). In contrast, necrosis involves swelling of organelles, loss of plasma membrane integrity and release of intracellular contents into the extracellular space (30). Mtb is able to continue growing inside necrotic macrophages, promoting lung inflammation and parenchymal injury (32). Mtb inhibits apoptotic cell death and promotes death of infected macrophages by necrosis (32). Roca et al. demonstrated the importance of ER-mitochondria signaling relay involving RyR and plasma membrane L-type Ca^2+^ channels for TNF-mediated necrosis of Mtb-infected macrophages in a zebrafish model of TB. Dantrolene reduced TNF-induced necrotic death of Mtb-infected macrophages by more than 50% by attenuating RyR activity and the surge in cytosolic Ca^2+^ that normally precedes necrosis, attesting to its potential as a HDT for TB (21). Inhibition of RyR activity with dantrolene has also been shown to promote autophagy (33), although this has not been demonstrated in Mtb-infected macrophages.

#### Resveratrol

Resveratrol (3, 5, 4′‐trihydroxystilbene) is a natural polyphenol produced by plants including grapes and berries and widely used as a food supplement (34, 35). It interacts with and modulates the activity of at least 20 mammalian proteins including CRAC channels and VGCCs (36). Resveratrol attenuates Mtb-induced inflammatory responses and enhances elimination of Mtb in macrophages, at least in part by upregulating the expression of host sirtuin 1 (37–39). Sirtuin 1 is a nicotinamide adenine dinucleotide (NAD^+^)-dependent deacetylase that deactivates ReLA, the p65 subunit of nuclear factor kappa B (NF-κB) (40). NF-κB is a transcription factor important in the maturation of dendritic cells, M1 polarization of macrophages, differentiation of Th0 cells to the Th1 phenotype and expression of pro-inflammatory cytokines including IL-1, IL-8 and TNF-α (41, 42). Mtb down-regulates expression of host sirtuin 1 in monocytes/macrophages, in mouse models of TB and in TB patients with active disease, leading to overexpression of NF-κB (40). While NF-κB expression is generally associated with anti-TB responses, NF-κB also inhibits apoptosis and autophagy, two of the pathways most effective at eliminating intracellular Mtb (43). Recently, Cheng et al. reported a log fold decrease in bacteria load after treating THP-1 cells infected with W148, an MDR-TB strain, with resveratrol for 3 days (40).

#### Verapamil

Verapamil is an L-type calcium channel (LTCC) blocker widely used for the treatment of hypertension, angina and abnormal heart rhythms. LTCCs are a subfamily of VGCCs, and are expressed on the plasma membrane of most cell types. Ca^2+^ currents through LTCCs inhibit release of ER calcium stores in macrophages, thus attenuating Ca^2+^-dependent signaling processes including macrophage activation (44). A previous study demonstrated that Mtb induces up-regulation of VGCCs in macrophages and dendritic cells to circumvent immune responses (44). Inhibiting LTCC currents in Mtb-infected macrophages with verapamil increases the concentration of Ca^2+^ in the cytosol, leading to upregulation of autophagy and Mtb killing (45–47).

The LTCC blockers verapamil and nifedipine also modulate iron metabolism by mobilizing iron from tissues, reducing intra-macrophage iron concentration and enhancing urinary iron excretion (48, 49). Iron is a cofactor in numerous biochemical reactions and is an essential nutrient for growth, replication and pathogenicity of many intracellular pathogens including Mtb (50). Further, prolonged iron overload promoted insulin resistance in skeletal muscle cells *in vitro* and *in vivo* in a mouse model of iron overload by inhibiting mTORC1 activation on autolysosomes and interfering with autophagic lysosomal regeneration (51). Therefore, by limiting iron availability, LTCC blockers promote a key pathway to enhance host resistance and clearance of intracellular pathogens such as Mtb.

In addition to enhancing host mycobacterial responses, Verapamil is bactericidal to both replicating and non-replicating Mtb in broth (52). It also inhibits both host and bacterial efflux pumps and is synergistic with RIF and isoniazid (INH) in broth, in macrophage cultures and in mouse models of TB (17, 52, 53). Gupta and colleagues reported that supplementing standard TB therapy with verapamil yielded an extra 1.15 log CFU reduction in pulmonary bacterial load in a murine model of TB (54).

#### Progress Towards Clinical Use of Calcium Channel Blockers as Anti-Tuberculosis Agents

While evidence from *in vitro* and animal models of TB indicates that calcium channel blockers have anti-TB activity, there has been no progress in transitioning from pre-clinical findings to clinical practice. The use of dantrolene as an anti-TB agent could prove challenging due to its numerous adverse effects which include muscle weakness, sedation, visual disturbances and hallucinations (55). Compared to dantrolene, verapamil and resveratrol are generally well tolerated and would be the preferred compounds for repurposing as anti-TB agents. Therefore, there is need for human clinical trials to assess the efficacy of verapamil and resveratrol as clinically relevant adjunct HDTs for TB.

### Potassium Channel Blockers

While Ca^2+^ directly modulate antimycobacterial responses, potassium (K^+^), sodium (Na^+^) and chloride (Cl^-^) ions primarily modulate macrophage responses by modulating Ca^2+^ currents.

K^+^ currents promote autophagy and other anti-TB responses in Mtb-infected macrophages (28). K^+^ is more abundant in the cytosol than the ECF while Ca^2+^, Na^+^ and Cl^-^ are more abundant in the ECF than the cytosol. This creates an overall negative charge inside the cell relative to the ECF. The electrochemical gradient between the cytosol and ECF facilitates movement of calcium into the cytosol during macrophage activation. Outward K^+^ currents help sustain Ca^2+^ entry and macrophage activation by preventing plasma membrane (PM) depolarization and maintaining an electrical gradient between the ECF and cytosol (56, 57). Several K^+^ channel antagonists have shown promise as potential anti-TB drugs, but most appear to promote macrophage anti-TB responses by mechanisms remote from ion channel blockade. Such compounds include chloroquine, ketoconazole and clofazimine.

#### Chloroquine

Chloroquine has been in use as an antimalarial agent for over 5 decades. It also suppresses the activity of mammalian delayed rectifier K^+^ channels (Kv1.3) in leukocytes and lymphocyte production of pro-inflammatory cytokines (58). Chloroquine has anti-inflammatory and immunomodulatory properties and is used to treat autoimmune diseases including rheumatoid arthritis and systemic lupus erythematosus. In addition, chloroquine has engendered interest as a potential HDT against several viral diseases.

Mishra and colleagues observed a modest reduction in growth of intracellular Mtb following exposure to chloroquine alone, but a five-fold increase in the activity of INH when the two compounds were used together (59). The combination of chloroquine with INH eliminated Mtb within 8 weeks in a murine model of TB, while INH alone only reduced bacterial load by 2 logs during the same timeframe. Additionally, chloroquine eradicated drug-tolerant Mtb, ameliorated lung pathology and reduced post-treatment TB relapse in *in vivo* mouse models of TB (59).

Chloroquine enhances the activity of INH through at least two divergent pathways. First, it inhibits phagosome acidification, thus reducing redox-induced Mtb drug tolerance (59). Second, chloroquine increases the intramacrophage concentration of INH by inhibiting the activity of p-glycoprotein and breast cancer resistance protein-1 (BCRP-1) (60, 61). P-glycoprotein and BCRP-1 are mammalian efflux pumps that are overexpressed in Mtb infected macrophages, where they extrude anti-TB drugs into the ECF, thus protecting the intracellular bacteria from the antibiotics (62). Together, these studies suggest potential for repurposing chloroquine to shorten the duration of current TB treatment and to achieve relapse-free cure.

#### Ketoconazole

Ketoconazole is an azole antifungal used to treat cutaneous and systemic fungal infections. It kills fungi by inhibiting synthesis of ergosterol, an essential component of the fungal PM (63). Ketoconazole also inhibits the activity of voltage-gated potassium channels (K_v_1.5, K_v_11.1) and other mammalian proteins (63, 64).

The azole class of antifungals has been reported to possess anti-TB activity. Byrne and colleagues reported that ketoconazole inhibited growth of Mtb in broth (12). Furthermore, they observed a 3.42 log CFU reduction in bacterial load in lungs of Mtb-infected mice that were treated with ketoconazole-RIF-INH-pyrazinamide (PZA), and a 3.08 log CFU reduction in mice that were treated with RIF-INH-PZA, indicating that ketoconazole is synergistic with current first-line anti-TB drugs (12). Ketoconazole enhances the activity of anti-TB drugs, at least in part, by inhibiting pregnane X receptor (PXR), a promiscuous ligand-dependent transcriptional factor that is activated by steroid and xenobiotic agents (65, 66). PXR modulates expression of mammalian drug efflux and metabolism genes and reduces the efficacy of rifamycins against intracellular Mtb (67). The role of ketoconazole in the treatment of TB should be explored further.

#### Clofazimine

Clofazimine is a riminophenazine dye that is used as a first line agent in the treatment of leprosy, in combination with RIF and dapsone (68). It kills mycobacteria by disrupting multiple physiological processes, including respiration and K^+^ transport across the PM (68). It was originally developed as an anti-TB drug more than five decades ago, but proved to be inferior to RIF and INH (68). The use of clofazimine to treat TB was revisited recently, and clofazimine has been listed as a second line anti-TB agent (2). The efficacy of various clofazimine-containing regiments against MDR-TB is currently being assessed in the BEAT TB, endTB-Q and TB-PRACTECAL clinical trials (2). Furthermore, pre-clinical studies show that clofazimine could reduce the duration of treatment of drug-susceptible TB. Tyagi and colleagues successfully sterilized Mtb-infected mice with a 3-month course of clofazimine-RIF-INH-PZA-ethambutol (EMB), but achieved a similar outcome with RIF-INH-PZA-EMB after treatment for 5 months (69). CLO-FAST, a phase 2 clinical trial is currently assessing the efficacy of a 3-month anti-TB regimen containing clofazimine and rifapentine against drug-susceptible TB (2).

In addition to its direct antimycobacterial activity, clofazimine has recently been shown to enhance host antimycobacterial responses by inhibiting mammalian Kv1.3 K^+^ channels, which are highly expressed on effector memory T (Tem) lymphocytes (70, 71). Singh and colleagues demonstrated that inhibition of Kv1.3 channels on Tem cells by clofazimine during BCG vaccination in mice enhanced vaccine efficacy by promoting selective expansion of central memory T (Tcm) cells, a T-cell subset that is associated with protective anti-TB responses. Mice that received clofazimine also exhibited significantly enhanced resistance against TB (71). These reports suggest Kv1.3 K^+^ channel blockade as a promising approach to enhance BCG vaccine efficacy in humans.

#### Progress Towards Clinical Use of Potassium Channel Blockers as Anti-Tuberculosis Agents

While clofazimine has now been adopted as an anti-TB agent, there has been no progress towards bringing ketoconazole and chloroquine into clinical use against TB. Azole antifungals such as ketoconazole and fluconazole are commonly used to treat concomitant candida or cryptococcal infections in HIV-infected TB patients but the impact of azole treatment on TB outcomes has not been assessed. Ketoconazole is generally well tolerated, but can cause serious adverse effects such as hepatotoxicity (72). Rifampicin, isoniazid and pyrazinamide can also cause drug-induced hepatitis (73), therefore, concomitant use of these anti-TB drugs with azole antifungals may increase the risk of hepatotoxicity. The safety of ketoconazole when used as adjunct treatment for TB requires further investigation.

### Sodium Channel Blockers

There is paucity of data on the role of Na^+^ channels in anti-TB responses. However, opening of PM Na^+^ channels leads to influx of Na^+^ into the cytosol down its chemical gradient, thus reducing the electrical gradient between the ECF and cytosol. This reduces the driving force for Ca^2+^ entry. Most PM Na^+^ channels may therefore inhibit host antimycobacterial responses, and several Na^+^ channel antagonists including ambroxol, carbamazepine and valproic acid promote host anti-TB responses (16, 19).

#### Ambroxol

Ambroxol is an inhibitor of voltage-gated sodium channel (Na_v_) 1.8 and is primarily used as a mucolytic agent (74). It is a potent inducer of autophagy and has garnered interest as a potential therapeutic agent to hasten degradation of misfolded proteins in proteinopathies including Parkinson’s disease and primary alveolar proteinosis (75, 76). It has no direct antimycobacterial activity (19, 53), but it induces dose-dependent autophagic control of Mtb *in vitro* and *in vivo* and promotes mycobacterial killing in Mtb-infected primary mouse macrophages (19). Additionally, Choi and colleagues observed that ambroxol potentiated the antimycobacterial activity of rifampicin in a murine TB model, resulting in a three-fold decrease in bacterial load in mice treated with ambroxol and RIF relative to mice treated with RIF alone (19). Ambroxol warrants further evaluation as a HDT to augment and enhance the efficacy of current chemotherapy for TB in humans.

#### Carbamazepine

Carbamazepine is used to treat epilepsy, schizophrenia and bipolar disorder. It inhibits the activity of Na_v_ 1.5, thus indirectly inhibiting the uptake of inositol through Na^+^-dependent inositol transporters on the PM (16). Inositol is a carbocyclic sugar upstream to biosynthesis of inositol-1,4,5-trisphosphate (IP_3_), a lipid second messenger that activates IP_3_Rs on ER. Blockade of inositol uptake by carbamazepine therefore reduces cytosolic levels of IP_3_, leading to decreased Ca^2+^ release from the ER and upregulation of autophagy (16). Treatment of MDR-TB-infected mice with carbamazepine for 30 days resulted in a ten-fold decrease in pulmonary bacterial load, improved lung pathology and stimulated adaptive immunity. This was achieved through induction of autophagic killing of intracellular Mtb, mediated by cellular depletion of inositol and independent of mTOR (16).

#### Valproic acid

Valproic acid is an inhibitor of Na_v_s, and is used to treat epilepsy, migraine and bipolar disorder (77, 78). It is active against Mtb in broth through mechanisms that have not been fully elucidated (20). Rao and colleagues observed a 1.5 log CFU reduction in bacterial load following treatment of intracellular Mtb with valproic acid or INH, and a 2 log CFU reduction when the two drugs were used together (20). Like carbamazepine, valproic acid promotes autophagy by interfering with biosynthesis of IP_3_ (79). In addition, it inhibits host histone deacetylase 1 (HDAC1), a protein that is usually upregulated in Mtb-infected macrophages (80, 81). HDACs suppress gene expression by promoting chromatin packaging, thus making a segment of DNA inaccessible to the cellular transcription machinery (82). Upregulating HDAC1 in Mtb-infected macrophages reduces expression of IL-12β, a cytokine important in the initiation of Th1 responses (80). Therefore, repurposing of carbamazepine and valproic acid as adjunct HDTs to enhance intracellular killing of Mtb by current anti-TB drugs should be explored as a treatment option for human TB.

#### Progress Towards Clinical Use of Sodium Channel Blockers as Anti-Tuberculosis Agents

The use of ambroxol, carbamazepine and valproic acid against TB has not been tested in a clinical trial setting. Carbamazepine and valproic acid are used widely as treatment for epilepsy in low- and middle-income countries where the burden of TB is high. However, the impact of concomitant use of sodium channel blockers and anti-TB treatment on TB outcomes has not been evaluated. Furthermore, both carbamazepine and valproic acid have been shown to cause hepatotoxicity in some individuals (83, 84), but there are no clinical trial data on the safety of carbamazepine and valproic when used in combination with existing anti-TB drugs.

### Other Ion Channel Blockers

#### Fluoxetine

Fluoxetine is a selective serotonin reuptake inhibitor (SSRI) and is primarily used as an anti-depressant. However, fluoxetine also has antiviral, antibacterial and immunomodulatory properties (85–87). In addition to inhibiting the uptake of serotonin into pre-synaptic neurons, fluoxetine modulates the activity of VGCCs, K2P, Na_v_s and 5-hydrotryptamine 3 (5-HT3) (88–90).

Schump and colleagues observed a 50% reduction in growth of intracellular Mtb following treatment with fluoxetine, even though it had limited activity against Mtb in broth (91). Several mechanisms for this have been described, including accumulation inside macrophages and induction of autophagy (15, 91). In another study, Stanley and colleagues demonstrated that fluoxetine promoted secretion of TNF-α, induced autophagy and inhibited growth of intracellular Mtb by 75% in J774 cells (15). These observations merit evaluation of the anti-TB activity of fluoxetine in clinical studies of human TB.

#### Phenothiazines

Phenothiazines are a large group of heterocyclic molecules most widely used as antipsychotics and antihistamines due to their ability to modulate dopamine signaling (92). Most phenothiazines bind to and modulate the activity of multiple mammalian proteins, including ligand-gated ion channels, ion pumps, G protein-coupled receptors and efflux pumps (92, 93).

The antimycobacterial properties of phenothiazines have been known for decades, but they were overshadowed by the current first line anti-TB compounds, to which they are inferior (7). However, the rise of MDR-TB has rekindled interest in the use of phenothiazines against Mtb. Most phenothiazines are active against extracellular Mtb at concentrations that cannot be achieved safely *in vivo*. However, they are generally active against intracellular Mtb at much lower concentrations (94). Some phenothiazines are concentrated by macrophages to at least 10 times their plasma concentrations, which may partly explain their potency against intracellular Mtb (94–96). Thioridazine, chlorpromazine, promethazine, methyldiazine and trifluoperazine are among the phenothiazines that have shown the most promise as potential anti-TB agents.

Thioridazine was once a popular drug for schizophrenia and psychosis but has largely been replaced by the newer generation of neuroleptics. It kills extracellular Mtb by disrupting ATP synthesis (17, 97). Machado and colleagues demonstrated that thioridazine promotes acidification of Mtb phagosomes, and reported an 88% increase in Mtb killing by thioridazine-treated macrophages (17). As thioridazine has multiple eukaryotic protein targets, the mechanism through which it promotes phagosome acidification has not been elucidated. However, one possible explanation is that it inhibits the efflux of ions from the phagolysosome, leading to indirect acidification (98).

In addition, thioridazine inhibits mycobacterial drug efflux systems, reduces resistance levels of different strains of MDR-TB to first and second-line anti-TB agents, and hastens clearance of drug-susceptible Mtb by first-line anti-TB agents (17, 95, 99). Dutta and colleagues were able to sterilize lungs of mice infected with drug-susceptible Mtb with a 4 month course of thioridazine-RIF-INH-PZA, but achieved the same with RIF-INH-PZA in 5 months (95).

#### Progress Towards Clinical Use of Other Ion Channel Blockers as Anti-Tuberculosis Agents

Fluoxetine and phenothiazines are currently not used as anti-TB drugs in clinical practice. However, thioridazine is relatively well tolerated, and has received more attention as a potential anti-TB agent than any other phenothiazine. There is need for clinical trials to determine the efficacy of thioridazine as part of anti-TB treatment regimens in humans.

## Summary and Concluding Remarks

Ion channel blockers have the potential to contribute to the treatment of TB to reduce morbidity and mortality from the disease. Their ability to enhance the activity of first-line anti-TB drugs could help hasten clearance of Mtb from lungs of individuals with pulmonary TB disease, reduce transmission of infection, emergency of drug-resistant mutants and relapse rates. The ideal host-directed therapeutics for TB should potentiate the immune system’s antimycobacterial defenses while preventing excessive inflammation and tissue injury. In addition to enhancing clearance of Mtb, ion channel blockers generally attenuate host inflammatory responses and may reduce tissue injury in TB patients. In the absence of a single agent that can eliminate Mtb, combination therapy will remain the mainstay of TB treatment. While current drug combinations are designed to maximize clearance of Mtb by targeting the pathogen, ion channel blockers could enhance bacillary clearance by targeting both the pathogen and the host immune response. The reduction in the risk of active TB associated with the use of dihydropyridine calcium channel blockers is a cause for optimism and may pave the way for clinical trials of ion channel blockers as adjunct treatment for human TB.

## Author Contributions

SM-N wrote the manuscript with input from HM and DT. All authors contributed to the article and approved the submitted version.

## Funding

HM and KJ are supported by African Research Leader Awards MR/P020526/1 and MR/T008822/1, respectively, jointly funded by the UK MRC and DFID under the MRC/DFID Concordant agreement. HCM is also supported by BMGF and NIH through grant numbers OPP1108452 and RO1AI155319, respectively. Wellcome Trust Core Funding Award number 206545/Z/17/Z supports the Malawi Liverpool Wellcome Trust Clinical Research Programme.

## Conflict of Interest

The authors declare that the manuscript was written in the absence of any commercial or financial relationships that could be construed as a potential conflict of interest.
